# Association between hospital participation in Medicare Shared Savings Program and hospital use of robotic surgical approach

**DOI:** 10.1007/s00464-025-11656-x

**Published:** 2025-03-21

**Authors:** Katia Noyes, Brooks Harmon, Joseph D. Boccardo, Ajay A. Myneni, Heather M. Link, David Abramowitz, Aaron B. Hoffman, Steven D. Schwartzberg

**Affiliations:** 1 Division of Health Services Policy and Practice, Department of Epidemiology and Environmental Health, School of Public Health and Health Professions, University at Buffalo, 270 Farber Hall, Buffalo, NY 14214 USA; 2 Department of Surgery, Jacobs School of Medicine and Biomedical Sciences, University at Buffalo, Buffalo, NY USA; 3 Department of Biostatistics, School of Public Health and Health Professions, University at Buffalo, Buffalo, NY USA; 4 Maternal Fetal Medicine Center, John R. Oishei Children’s Hospital, Buffalo, NY USA; 5 Department of Urology, Jacobs School of Medicine and Biomedical Sciences, University at Buffalo, Buffalo, NY USA; 6 Department of Surgery, Methodist Dallas Medical Center, Dallas, TX USA

**Keywords:** Affordable Care Act (ACA), Accountable care organization (ACO), Medicare Shared Savings Program (MSSP), Robotic surgery, Hospital rankings

## Abstract

**Background:**

In 2012, Medicare introduced Shared Savings Program (MSSP) accountable care organizations (ACO) model to improve the value of health care services as a part of the national comprehensive Accountable Care Act. While the effect of the MSSP on primary care has been extensively analyzed, little is known about the effect of the MSSP on cost and quality of surgical care, in particular the use of high-cost robotic surgical modalities. Hospitals routinely market robotic procedures as an indicator of high quality, despite limited evidence of their clinical value. This study examines the relationship between hospital participation in the MSSP and use of robotic surgery.

**Methods:**

We conducted a retrospective analysis using 2016–2019 publicly available data on hospital MSSP participation and use of robotic-assisted procedures in New York State. Using bivariate and multivariate approaches, we identified hospital characteristics associated with the use of robotic technique and hospital quality.

**Results:**

Of the 157 general hospitals in NYS, 83 (53%) offered robotic surgery and 73 (47%) participated in the MSSP. MSSP-affiliated hospitals were more selective in the type of robotic procedures than non-MSSP hospitals, favoring procedures with stronger evidence-base such as prostatectomies. Hospitals that performed robotic surgery selectively had significantly lower spending per patient (*p* = 0.04). Higher volume of robotic procedures correlated with higher hospital ranking.

**Conclusions:**

MSSP participation is associated with more selective use of robotic procedures and lower hospital spending. More research is needed to understand the relationship between hospital investments in quality improvement, use of robotic surgery and hospital performance rankings.

**Supplementary Information:**

The online version contains supplementary material available at 10.1007/s00464-025-11656-x.

Since its implementation in 2012, Medicare Shared Savings Program (MSSP) accountable care organizations (ACO) generated $3.618 billion in gross savings or $1.7 billion after accounting for shared savings payments. The MSSP savings came primarily from improving care coordination for patients with chronic conditions and reducing preventable emergency visits and hospitalizations. The MSSP implementation has supported the shift toward value-based payment and integrated care-delivery models [1] Under the MSSP model, physicians, hospitals, and other health care providers are encouraged to form a partnership to deliver high value care for a specific population of Medicare patients [2–4]. The providers in each partnership are responsible for managing the healthcare costs and achieving high quality outcomes of their assigned beneficiaries [2]. If an ACO adheres to predefined quality and financial performance benchmarks, they share the savings generated by the Medicare program [5]. The specific approaches to reduce costs, improve quality and maximize patient experience, however, vary greatly among ACOs, and within ACOs—between different service lines and specialties.

Most of the published literature on ACO impact is focused on early phases of ACO dissemination that was characterized by a significant selection of quality-driven facilities into the ACO model and low participation of physician specialists in ACOs activities. As a result, the evidence on the effects of ACOs on primary care, hospital admissions and readmissions is extensive [6–13]; however, little is known about how and whether MSPP model impact surgical and other specialty care [14, 15].

Robotic-assisted surgery was designed to increase precision, flexibility and control during surgical procedures. The robotic approach initially demonstrated its superiority over open surgery for prostate cancer (i.e., radical prostatectomy), and since diffused to many other procedures in urology, general and gynecologic surgery [16–21]. Despite the rapid uptake of robotic surgery, with substantially higher institutional costs than other surgical alternatives, the advantage of robotic-assisted approach has not been demonstrated consistently across all surgical procedures [22, 23]. Despite these shortcomings, many hospitals strongly support the use of robotic surgery as a sign of technologic innovations, and to attract patients in competitive hospital markets [24].

Inpatient and ambulatory surgical services offer a significant opportunity for improving value, as payments for surgical care constitute nearly half of Medicare expenditures[25] and nearly a third of total health care expenditures in the US [26, 27]. Still, previous literature identified no substantive changes in quality or cost of surgical care in ACO-affiliated hospitals compared to non-ACO facilities, in sharp contrast with improvements in primary care and chronic disease management [7, 15, 28]. Modi et al. (2020) reported no differences in the use of new surgical technologies between ACO and non-ACO hospitals indicating that ACO do not restrict use of surgical innovations [29].

Herein, using more recent data on MSPP participation, we examined the use of robotic-assisted surgical approaches for indications with various strength of supportive evidence in urology, gynecology and general surgery [30, 31]. We also assessed whether CMS hospital quality metrics (CMS Five-Star Quality Rating, Medicare Spending Per Beneficiary (MSPB), inpatient revenue, hospital Medicare discharges, and total hospital discharges based on overall hospital performance and patient satisfaction) are aligned with markers for surgical quality, specifically the use of robotic-assisted surgical procedures.

## Materials and methods

### Data sources

Hospitals ACO status in a given year was determined from the MSSP annually reports that list all participating ACOs alongside their associated participants [32]. Hospital quality metrics (CMS Five-Star Quality Rating and Medicare Spending Per Beneficiary (MSPB)) were obtained from Hospital Compare, CMS online open-use database on all Medicare-certified hospitals to assist consumers in identifying care value [33]. The CMS also maintains provider cost report data Healthcare Cost Reporting Information System (HCRIS)[34] that we used to collect data on hospital inpatient revenue, Medicare discharges, and total discharges.

To determine hospital surgical volume for robotic-assisted and other minimally invasive procedures (Supplemental Table 1), we used the NYS Statewide Planning and Research Cooperative System (SPARCS) database. SPARCS is an all-payer data reporting system that includes patient characteristics, diagnoses, and services for all hospital admissions in NYS [6].

### Hospital quality indicators

#### CMS five-star quality rating

The Five-Star Quality Rating ranges from a minimum of one to a maximum of five stars. It is designed to aggregate quality-related data into a single measure and help patients compare overall hospital quality. In estimating these ratings, CMS considers five categories of data: mortality, safety of care, readmissions, patient experience, and timely and effective care [35].

#### Medicare spending per beneficiary (MSPB)

MSPB is used to represent how spending for each Medicare patient varies across U.S. hospitals. Rather than present spending as a monetary value, CMS uses a ratio to represent MSPB. The ratio is calculated as the amount of spending per beneficiary during an episode of care divided by the national median amount Medicare spends. Payments used to calculate MSPB include Medicare Part A and B payments related to each episode of care, including payments made three days before an inpatient stay, the inpatient stay itself, and the 30-day period following discharge. To control for variation resulting from geographical differences, these payments are risk-adjusted and price-standardized [33].

#### Inpatient revenue

CMS defines hospital inpatient revenue as the total inpatient routine services and inpatient ancillary services [33].

#### Medicare and total hospital discharges

CMS defines Medicare and total hospital discharges as the formal release of Medicare and all patients, including release due to death [33].

### Robotic and non-robotic surgical volume

From SPARCS hospital discharge data, we determine the volume of procedures performed using either the robotic-assisted or standard approach for cholecystectomy, gynecologic, urologic, and bariatric procedures using ICD and CPT codes (Supplemental Table 1).

### Covariates

We used Rural–Urban Continuum Codes (RUCC) classification and geographical location data from SPARCS to categorize hospitals into metropolitan (RUCC 1–3) and non-metropolitan (RUCC 4–9) [36]. Hospital teaching status was determined based on the CMS Open Payments database, a national disclosure program that publishes payments made by drug and medical device companies to health care providers, including teaching hospitals [37]. Number of hospital beds was obtained from the NYS Hospital Profiles, published by the NYS Department of Health [38]. All hospitals in NYS are not-for-profit.

### Study sample

**Fig. 1:**
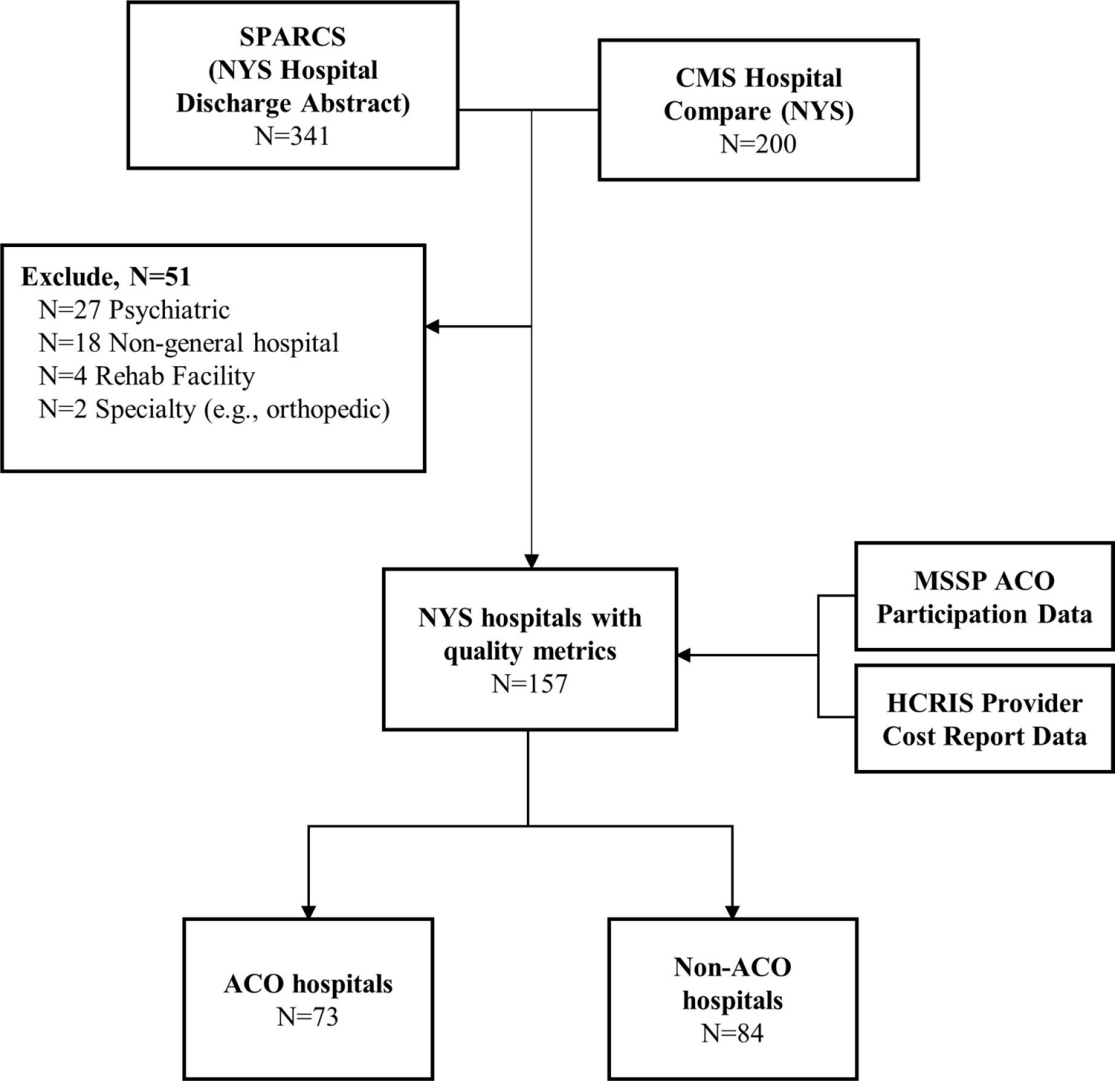
Flowchart of sample of MSSP ACO hospitals and non-ACO hospitals. Centers for Medicare and Medicaid Services (CMS) Hospital Compare datasets from the 2016–2019 were matched to data from the Statewide Planning and Research Cooperative System (SPARCS). After combining 200 hospitals from Hospital Compare and 341 unique facility names and CMS National Provider Identifiers (NPIs) from SPARCS, we excluded 51 hospitals and applied data from CMS MSSP ACO Participants data and from HCRIS provider cost reports to the final sample of 157 hospitals. Of these hospitals, 73 participated in the ACO model for at least one year and 84 did not participate at all

The study sample included NYS general hospitals listed in both Hospital Compare and SPARCS from 2016–2019. We limited the sample to facilities that performed at least one urologic, gynecologic or general surgery surgical procedure (*n* = 341; see Supplementary Table 1). We excluded psychiatric (*n* = 27), rehabilitation (*n* = 4), specialty hospitals (*n* = 2), and ambulatory surgical centers (*n* = 13). We also excluded any children’s hospitals (*n* = 1), military hospitals (*n* = 1), and birthing centers (n = 1), as well as hospitals that merged with another organization over the study period (*n* = 2) [39]. The final sample consisted of 157 hospitals that satisfied the inclusion criteria Fig. 1.

### Statistical analysis

For bivariate analysis, we used Fisher’s exact test or chi-square test, where appropriate, for categorical variables to compare the descriptive characteristics between different hospitals subgroups. Due to the hospital measurements being repeated over the study period, we used mixed effect models to analyze the effect of ACO participation, year of the study, bed size, hospital teaching status, and hospital geography on all CMS quality metrics. CMS Five-Star Rating was modeled using a logistic regression model, low rating (1–3) vs high rating (4–5). Since less than 5% of hospitals had a five-star rating, we combined four-star and five-star hospitals into one category. We analyzed MSPB, inpatient revenue, Medicare discharges, and total discharges using ANOVA where log-transformations were applied to inpatient revenue, Medicare discharges, and total discharges to normalize the residuals. Logistic regression models were also used to analyze the relationship between hospital robotic capability and their ACO status, average MSPB between 2016 and 2019, average total discharges between 2016 and 2019, average CMS Five-Star Quality Rating between 2016 and 2019, hospital teaching status, and hospital geography.

To determine the relationship between MSSP participation and the robotic-assisted procedure volume for each procedure type, we fitted negative binomial models with total procedure volume as the offset. These models were fitted with MSSP status plus average MSPB, average total discharge, average CMS Five-Star Quality Rating, hospital teaching status, and hospital geography. All statistical tests were two-sided, and alpha was set at 0.05. All analyses were conducted using SAS 9.4 (Cary, NC) [40].

## Results

### Characteristics of MSSP ACO and non-ACO hospitals

Of the 157 hospitals in our sample, 73 (46.5%) participated in the MSSP ACO model for at least one year between 2016 and 2019, while 84 (53.5%) did not participate in the MSSP ACO in any year. There were no statistically significant differences between MSSP ACO and non-ACO hospitals in NYS by teaching status, hospital geography, hospital size, hospital robotic surgery capabilities or hospital quality metrics (Table 1).

**Table 1:** Select characteristics of MSSP ACO and Non-ACO hospitals

Variable	MSSP ACO hospitals^1^ *N* = 73 *N* (%)	Non-ACO hospitals *N* = 84 *N* (%)	*P*^*2*^
Hospital teaching status			
Teaching	43 (58.9)	54 (64.3)	0.49
Non-teaching	30 (41.1)	30 (35.7)	
Hospital geography^3^			
Metropolitan area	57 (78.1)	72 (85.7)	0.30
Non-metropolitan area	16 (21.9)	12 (14.3)	
Number of hospital beds			
< 50	9 (12.2)	6 (7.1)	
50–300	35 (47.3)	47 (56.0)	0.46
> 300	29 (39.7)	31 (36.9)	
Hospital robotic surgery capability^4^	44 (60.3)	39 (46.4)	0.08
Cholecystectomy	38 (52.1)	36 (42.9)	0.25
Gynecologic	39 (53.4)	38 (45.2)	0.31
Genitourinary	39 (53.4)	37 (44.1)	0.24
Bariatric	37 (50.7)	36 (42.9)	0.33
Hospital quality metrics [mean (SD)]			
Average rating	2.37 (0.81)	2.13 (0.96)	0.06
Average MSPB	0.97 (0.06)	1.00 (0.07)	0.05
Average discharge (medicare)	3,352 (4,275)	3,656 (3,482)	0.19
Average discharge (ALL)	13,443 (17,487)	13,591 (14,915)	0.57

*MSSP* Medicare Shared Savings Program, *ACO* accountable care organization, *MSPB* Medicare spending per beneficiary

^1^The sample of MSSP ACO hospitals include hospitals that participated in a MSSP ACO for at least one year during 2016–2019. Non-ACO hospitals did not participate in any years from 2016 to 2019; ^2^*P*-values from Fisher’s Exact or Chi-Square tests. *P* values shown in bold font indicate statistically significant values; ^3^Metropolitan Area is indicated by Rural–Urban continuum codes (RUCC) 1 through 3, whereas Non-Metropolitan Area is indicated by RUCCs 4 through 9; ^4^Hospital robotic surgery capability was defined as hospitals that performed at least one of the following procedures with the robotic-assisted approach: cholecystectomy, gynecologic, genitourinary, or bariatric

### Characteristics of hospitals based on availability of robotic platform

We identified 83 (52.9%) hospitals that performed at least one robotic-assisted procedure of any type (Table 2). Hospitals with robotic capabilities (52.9%) were more likely to be large teaching hospitals located in metropolitan areas (*p* < 0.01) (Fig. 1).

**Table 2:** Select characteristics of MSSP ACO and Non-ACO hospitals by robotic surgery capability

Variable	Hospitals with no robotic surgeries *N* = 74 (47.1%) N (%)	Hospitals with at least one type of robotic surgery *N* = 83 (52.9%) N (%)	*P*^1^	Hospitals with all four types of robotic surgeries *N* = 65 (78.3%) N (%)	Hospitals with 1–3 types of robotic surgeries *N* = 18 (21.7%) N (%)	*P*^1^
Hospital teaching status			** < 0.01**			
Teaching	37 (50.0)	60 (72.3)		49 (75.4)	11 (61.1)	0.25
Non-teaching	37 (50.0)	23 (27.7)		16 (24.6)	7 (38.9)	
Hospital geography^2^			** < 0.01**			**0.04**
Metropolitan area	52 (70.3)	77 (92.8)		62 (95.4)	15 (83.3)	
Non-metropolitan area	22 (29.7)	6 (7.2)		3 (4.6)	3 (16.7)	
ACO at least one year			0.08			**0.03**
Yes	29 (39.2)	44 (53.0)		30 (46.2)	14 (77.8)	
No	45 (60.8)	39 (47.0)		35 (53.8)	4 (22.2)	
Number of hospital beds			** < 0.01**			**0.02**
< 50	14 (18.9)	1 (1.1)		0 (0)	1 (5.6)	
50–300	42 (56.8)	40 (48.2)		28 (43.1)	12 (66.7)	
> 300	18 (24.3)	42 (50.6)		37 (56.9)	5 (27.8)	
Hospital robotic surgery capability^3^	0 (0)	83 (100)		65 (100.0)	18 (100)	** < 0.01**
Cholecystectomy	0 (0)	74 (89.2)		65 (100.0)	9 (50.0)	** < 0.01**
Gynecologic	0 (0)	77 (92.8)		65 (100.0)	12 (66.7)	** < 0.01**
Genitourinary	0 (0)	76 (91.6)		65 (100.0)	11 (61.1)	** < 0.01**
Bariatric	0 (0)	73 (88.0)		65 (100.0)	8 (44.4)	** < 0.01**
Hospital quality metrics [mean (SD)]						
Average rating	2.25 (0.93)	2.23 (0.88)	0.92	2.19 (0.91)	2.41 (0.76)	0.27
Average MSPB	0.983 (0.08)	0.987 (0.06)	0.58	0.995 (0.05)	0.958 (0.06)	**0.04**
Average discharge (Medicare)	1,807 (2,155)	5,156 (4,400)	** < 0.01**	5,774 (4,669)	2,841 (1,945)	**0.01**
Average discharge (ALL)	7,118 (9,788)	19,673 (18,476)	** < 0.01**	22,030 (19,684)	10,834 (8,787)	**0.01**

*MSSP* Medicare Shared Savings Program, *ACO* accountable care organization, *MSPB* Medicare spending per beneficiary

^1^*P*-values from Fisher’s Exact or Chi-Square tests. *P* values shown in bold font indicate statistically significant values; ^2^Metropolitan area is indicated by Rural–Urban Continuum Codes (RUCC) 1 through 3, whereas Non-metropolitan area is indicated by RUCCs 4 through 9; ^3^Hospital robotic surgery capability was defined as hospitals that performed at least one of the following procedures with the robotic-assisted approach: cholecystectomy, gynecologic, genitourinary, or bariatric

Hospitals that offered robotic-assisted alternatives for some but not all indications (21.7%) were more likely to be a part of an ACO (77.8 vs 46.2%, *p* = 0.03), were smaller than hospitals that offered robotic surgery for all indications (10,834 vs 22,030 discharges per year, *p* < 0.01) and had lower per beneficiary spending (*p* = 0.04). Of the hospitals in our sample, 77 (49.0%) at least one robotic-assisted gynecologic procedure, 76 (48.4%) at least one robotic-assisted urologic procedure, 74 (47.1%) performed at least one robotic-assisted cholecystectomy, and 73 (46.5.1%) at least one robotic-assisted bariatric procedure.

### Analysis of temporal trends in hospital performance by MSSP ACO status and robotic capabilities

In our analysis of time trends, we found that MSSP ACO participation was associated with lower per patient spending compared to hospitals not participating in the MSSP ACO model (Table 3). We also observed significant increases in per patient spending and overall inpatient revenue and decrease in hospital discharges over time (*p* < 0.01). Our analysis also revealed that hospitals with robotic capabilities and teaching hospitals in NYS spent more per Medicare beneficiary and have higher inpatient revenue and number of discharges.

**Table 3:** Effect of hospital factors on financial and quality performance^1^

Hospital factors	CMS five–star quality rating OR (95% CI)	MSPB OR (95% CI)	Inpatient revenue OR (95% CI)	Medicare discharges OR (95% CI)	Total discharges OR (95% CI)
ACO status					
No	Ref	**–**	**–**	**–**	**–**
Yes	1.09 (0.18, 6.63)	**− 0.02 (− 0.03, − 0.01)**	0.02 (− 0.07, 0.11)	− 0.01 (− 0.08, 0.06)	0.01 (− 0.06, 0.07)
Year					
2016	Ref	Ref	**–**	**–**	**–**
2017	0.91 (0.09, 9.03)	0.00 (− 0.00, 0.01)	0.03 (− 0.01, 0.07)	− 0.01 (− 0.04, 0.02)	0.00 (− 0.03, 0.03)
2018	0.88 (0.08, 9.19)	**0.01** **(0.00, 0.01)**	**0.08** **(0.04, 0.13)**	**− 0.04** **(− 0.08, − 0.01)**	− 0.02 (− 0.05, 0.01)
2019	1.70 (0.19, 15.25)	**0.01** **(0.01, 0.02)**	**0.08** **(0.04, 0.12)**	**− 0.10** **(− 0.13, − 0.07)**	**− 0.06** **(− 0.09, − 0.03)**
Number of beds		–	**–**	**–**	**–**
< 50	Ref	–	**–**	**–**	**–**
50–300	0.34 (0.02, 4.78)	0.04 (− 0.01, 0.09)	**1.76** **(1.32, 2.20)**	**1.37** **(0.98, 1.43)**	**1.57** **(1.25, 1.89)**
> 300	0.30 (0.01, 12.27)	0.05 (− 0.01, 0.10)	**3.04** **(2.51, 3.58)**	**2.07** **(1.59, 2.55)**	**2.51** **(2.11, 2.90)**
Hospital teaching status					
Non-teaching	Ref	–	–	–	–
Teaching	0.55 (0.05, 6.20)	**0.04** **(0.02, 0.07)**	**0.76** **(0.44, 1.07)**	**0.36** **(0.08, 0.64)**	**0.59** **(0.36, 0.82)**
Hospital geography					
Metro	Ref	–	–	–	
Non-metro	1.13 (0.11, 11.74)	**− 0.05** **(− 0.08, − 0.02)**	**− 0.44** **(− 0.79, − 0.10)**	− 0.06 (− 0.37, 0.25)	− 0.20 (− 0.46, 0.05)
Robotic capability					
No	Ref	–	–	–	–
Yes	1.55 (0.21, 11.53)	− 0.01 (− 0.03, 0.01)	**0.60** **(0.35, 0.86)**	**0.74** **(0.51, 0.96)**	**0.61** **(0.42, 0.80)**

*ACO*, Accountable care organization, *CI* Confidence interval, *CMS* Centers for Medicare and Medicaid Services, *MSPB* Medicare spending per beneficiary

^1^CMS five−star quality rating were analyzed using a proportional odds mixed model. These results are presented as odds ratios. MSPB, inpatient revenue, Medicare discharges, and total discharges were analyzed using ANOVA

Estimates shown in bold indicating statistical significance

### Multivariable analysis of the effect of ACO status and hospital quality metrics on hospital use of robotic surgery

**Fig. 2:**
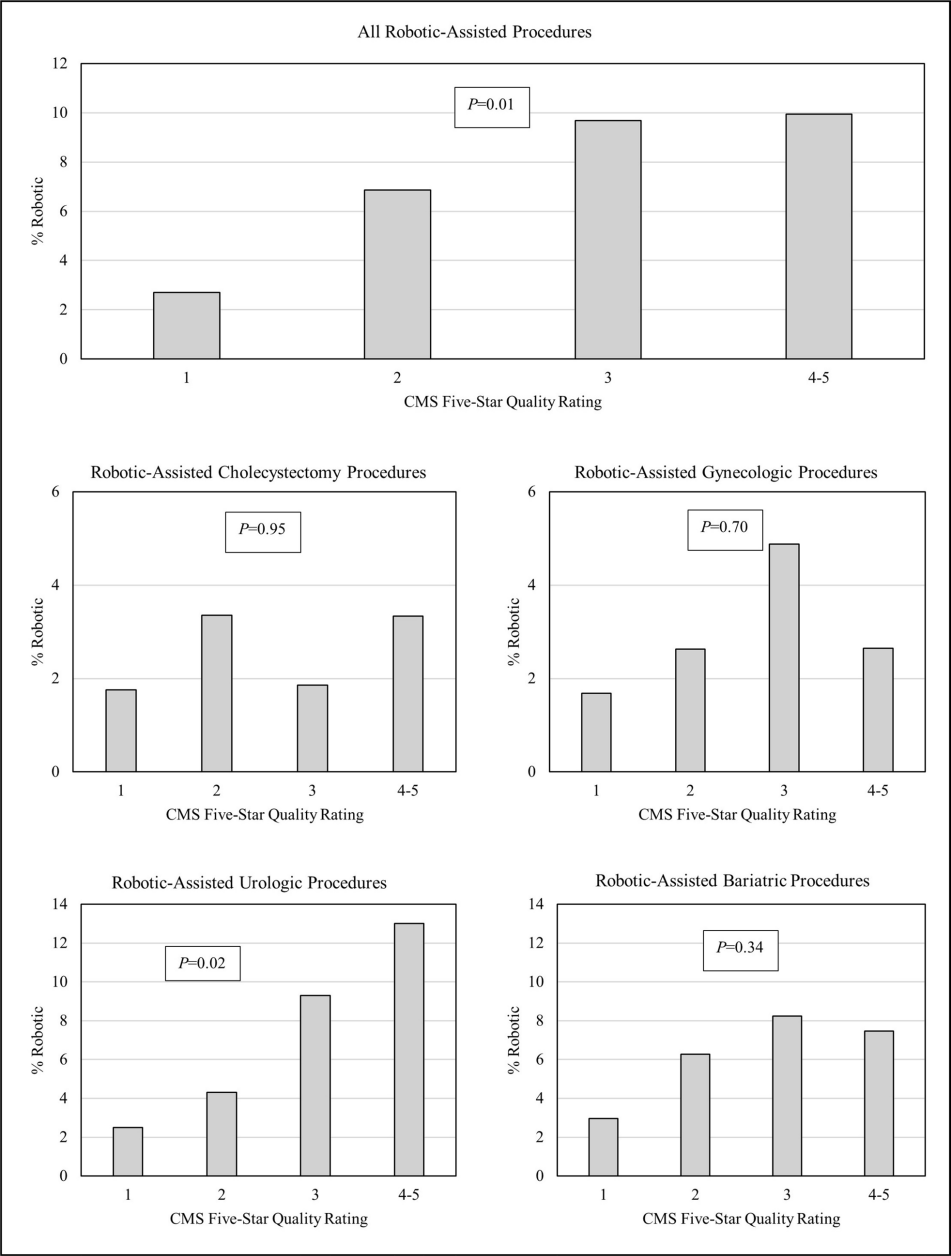
Percentage of inpatient procedures performed robotically by CMS Five-Star Quality. These bar charts illustrate the percentage of procedures performed using the robotic-assisted approach in relation to CMS Five-Star Quality Rating. We observed significant associations as ratings increased for robotic-assisted urologic procedures as well as all robotic-assisted procedures at the *P* < 0.05 level. These associations are also presented in Table 5. Abbreviations: *SPARCS* Statewide Planning and Research Cooperative System, *CMS*, Centers for Medicare and Medicaid Services, *ACO* Accountable care organization, *MSSP* Medicare Shared Savings Program, *NYS* New York State, *HCRIS* Healthcare Cost Reporting Information System

ACO-affiliated hospitals were significantly more likely to invest in robotic-assisted platforms than non-ACO facilities (OR 2.34 95% CI 1.05–5.20, Table 4a). Among hospitals that had robotic capabilities, being affiliated with an ACO was not significantly associated with the volume of robotic procedures performed, except for cholecystectomies (0.95 95% CI 0.36, 1.54, Table 4b). Hospital robotic volumes, especially for prostatectomies, were also positively correlated with hospital Five-Star ratings (Fig. 2, Table 5).

**Table 4:** Effect of MSSP ACO status on hospital characteristics

Procedure type	*N*^1^	OR^2^	95% CI
a. Effect of MSSP ACO status on hospital having robotic capability			
Robotic cholecystectomy	136	1.81	0.82–3.97
Robotic gynecologic	138	1.73	0.79–3.81
Robotic urologic	139	2.01	0.89–4.51
Robotic bariatric	132	2.22	0.88–5.61
Any robotic	139	**2.34**	**1.05–5.20**

	*N*^3^	*ß*^*4*^	95% CI
b. Effect of MSSP ACO status on robotic procedure volume. (ACO CAT ONLY)			
Robotic cholecystectomy	74	**0.95**	**0.36, 1.54**
Robotic gynecologic	77	0.14	− 0.33, 0.60
Robotic urologic	76	− 0.26	− 0.70, 0.18
Robotic bariatric	73	− 0.03	− 0.59, 0.53
Any robotic	83	0.05	− 0.34, 0.45

Estimates shown in bold indicating statistical significance. For odds ratio, the significance was determined based on 95% confidence intervals derived from multiple logistic regression model (a confidence interval that does not include 1 indicates a significant difference). For the beta coefficients, the significance was determined based on 95% confidence intervals derived from negative binomial model (a confidence interval that does not include 0 indicates a significant difference)

*MSSP* Medicare shared savings program, *ACO* Accountable care organization, *OR* Odds ratio, *CI* Confidence interval

^1^Number of hospitals included in the model by procedure type; ^2^Odds ratio and confidence intervals derived from multiple logistic regression models for each procedure type with CMS Five-Star Quality Rating, Medicare Spending Per Beneficiary (MSPB), total hospital discharges, hospital teaching status, and hospital geography as covariates; ^3^among hospitals with robotic capabilities. ^4^*ß*s and confidence intervals were derived from models of negative binomial for each procedure type

**Table 5:** Effect of hospital factors on robotic procedure volume

Procedure type	*N*^1^	*ACO* *ß* (95% CI)^2^ (> 0 y vs 0 y)	Average rating ß (95% CI)^2^	Average MSPB ß (95% CI)^2^	Average discharge ß (95% CI)^2^	Type academic ß (95% CI)^2^ (yes vs no)	RUCC metro^3^ ß (95% CI)^2^ (metro vs non-metro)
Robotic cholecystectomy	68	0.47 (− 0.20, 1.15)	− 0.18 (− 0.59, 0.23)	5.77 (− 1.36, 12.89)	0.00 (− 0.00, 0.00)	**− 1.20** **(− 2.16, − 0.23)**	− 0.77 (− 2.01, 0.48)
Robotic gynecologic	71	**0.58** **(0.07, 1.08)**	0.08 (− 0.21, 0.38)	3.71 (− 0.93, 8.34)	0.00 (− 0.00, 0.00)	− 0.15 (− 0.75, 0.44)	**1.52** **(0.49, 2.54)**
Robotic urologic	70	− 0.16 (− 0.60, 0.28)	**0.37** **(0.09, 0.65)**	− 0.63 (− 4.7, 3.49)	0.00 (− 0.00, 0.00)	− 0.03 (− 0.59, 0.52)	**1.33** **(0.41, 2.25)**
Robotic bariatric	67	− 0.18 (− 0.78, 0.42)	0.16 (− 0.22, 0.54)	5.31 (− 0.22, 10.8)	0.00 (− 0.00, 0.00)	0.42 (− 0.39, 1.23)	− 0.85 (− 2.34, 0.65)
Any robotic	76	0.19 (− 0.25, 0.62)	**0.29** **(0.03, 0.56)**	2.86 (− 1.16, 6.87)	0.00 (− 0.00, 0.00)	− 0.27 (− 0.81, 0.27)	0.29 (− 0.56, 1.15)

*MSSP* Medicare Shared Savings Program, *ACO* Accountable care organization, *RUCC* Rural–Urban continuum codes

Bold estimates indicate statistical significance

^1^Among hospitals with robotic capabilities; ^2^*ß* were derived from models of negative binomial for each procedure type; ^3^Metropolitan status based on RUCC, Metro regions were indicated by RUCC 1 through 3, whereas Non-metropolitan area is indicated by RUCCs 4 through 9

## Discussion

Our analysis of recent trends in the use of value-based surgical strategies in MSSP participating vs non-participating hospitals in NYS identified three noteworthy findings. First, MSPP-participating hospitals were more selective in the type of robotic procedures offered than non-participating hospitals, focusing on procedures with stronger evidence base such as prostatectomies. Second, hospitals that performed robotic surgery selectively had significantly lower spending per patient. Finally, higher volumes of robotic procedures correlated with higher hospital ranking.

Shortly after the MSSP began, Dupree and others concluded that ACOs place “little emphasis on value in surgical care” and instead focus on admissions and readmissions, care coordination for complex patients, and ED use [7]. Researchers have since indicated that MSSP participating healthcare organizations have largely failed to effectively engage with surgical care [14, 29, 41, 42]. During the same time, the use of robotic-assisted procedures experienced a near threefold increase, notwithstanding higher costs and inconclusive evidence of its superiority compared to the standard minimally invasive approach for many common surgical procedures [22, 23, 26, 30, 31, 43, 44]. When combined with the observation that an episode costs for robotic-assisted surgery could be as much as 25% cost of laparoscopy, robotically assisted surgical approach is often categorized as low-value care [15].

Despite these shortcomings, the robotic-assisted surgery market has grown steadily, driven by technological upgrades, increased patient demand for minimally invasive procedures, and ergonomic and visualization advantages for surgeons. The market for robotic-assisted surgery is expected to climb to $23 billion globally by 2030 [45]. Robotic-assisted surgery significantly enhances a healthcare organization’s reputation as a center of excellence and attracts patients seeking cutting-edge treatment options. Surgeons are drawn to a similar set of advantages, including enhanced safety monitoring tools and superior ergonomics, especially for community surgeons operating without an assistant surgeon, and boost to their professional reputation. For surgeons early in their careers, showing proficiency in robotic technology can be beneficial in affiliating with a hospital and in attracting patients.

Earlier studies noted comparable levels of adoption of newer surgical procedures between ACOs and non-ACOs [29] as well as the limited effect of ACOs on the use of low-value services [8, 14, 46]. Nearly half of all hospitals in our sample offered robotic surgical procedures, both among ACOs and non-ACOs. However, among hospitals with robotic capabilities, those that were affiliated with ACOs were more likely to only use robotic set-up for the interventions with established evidence base (i.e., prostatectomies) and higher volumes. Furthermore, our findings indicated that during 2016–2019, NYS ACO-affiliated hospitals had lower spending per patient than non-affiliated hospitals, with the subset of hospitals that used robotic surgery selectively having the lowest MSPB. We hypothesize that these savings were potentially due to cumulative effect of multiple value-enhancing strategies including Enhanced Recovery After Surgery (ERAS), standardization of procedures and protocols, guideline-concordant use of minimally invasive techniques, use of multidisciplinary teams, monitoring and analysis of data quality, and other quality improvement initiatives [47].

Despite the effects described above, the wide and persistent use of robotic-assisted procedures by both MSSP participating and non-participating hospitals, including even minor procedures, such as inguinal hernia repair, could be explained by many factors. First, the ACO-associated financial incentives (either penalties or savings) to shift care from volume-based to value-based models may not be strong enough to persuade healthcare providers to alter their behaviors. To that effect, little is known about how surgeons engage with and are reimbursed by ACOs. Hospitals and surgeons may also find it more advantageous to continue the use of high-margin procedures (i.e., robotically assisted surgery) to retain and improve surgical skills and team expertise necessary to perform complex robotic procedures [15, 29, 46, 48, 49]. In addition, MSSP ACOs have been “plagued by weak incentives” from the beginning, in part because of CMS’s benchmarking methodology, which was established based on historical spending during a baseline period and “rebased” to calculate shared savings [50]. ACOs that are successful in generating savings are confronted by ever-decreasing benchmarks, forcing value-focused hospitals to leave the ACO model [50].

Our analysis did not identify any significant differences between characteristics of MSSP ACO vs. non-ACO hospitals. This is in contrast with the previous studies that used national data that demonstrated that ACO hospitals were more likely to be urban and larger than non-ACOs [51]. However, in NYS, the county-level ACO penetration rate, defined as the proportion of Medicare fee-for-service beneficiaries assigned to an MSSP ACO, is highest in the rural counties [52]. In addition, although before 2016 rural hospitals were less likely to join ACOs, more than 50 ACOs with a considerable rural presence had joined the MSSP and the majority of new ACOs had been rural providers in 2016 [53]. Increases in overall NYS inpatient revenue and MSPB, on the other hand, could be the result of horizontal hospital consolidation and hospital size, given that over three-quarters of NYS hospitals already belong to or are affiliated with a larger health system [54].

Our findings offer recommendations for future policy. Going forward, it is critically important to consider care quality, impact of surgical costs on Medicare programs, and the overall effect of surgical care on hospital market share [25]. Inpatient surgical care consumes roughly 50% of hospital expenditures and 30% of overall health care costs [42]. These significant costs illustrate areas with great potential savings for Medicare. With surgeons not actively participating but only “passively absorbed” by the ACO model [9], we believe that to influence surgical care, CMS should determine new ways to engage surgeons and other specialists in value-based care and quality improvement programs [1, 55]. Given the ongoing transition to an older society and the coming surge in Medicare beneficiaries, which will only accentuate rising health care expenditures, it will become even more necessary to engage high-cost services providers (e.g., surgeons) in the conversation about value and quality in healthcare [56].

Our study has several limitations. First, our results may not be generalized to other states, especially states with for-profit hospitals. Second, we could not determine whether surgeons that performed the identified procedures were employed by MSSP ACOs or only had hospital privileges [25]. Third, we did not account for the variation in organization and staffing of the ACOs within our sample and have no information about whether or not a particular ACO used explicit strategies to improve value of surgical services [57]. Fourth, analysis presented here only considered hospital-based surgical procedures; more research is needed to understand the effect of MSSP on performance of the growing number of ambulatory surgical centers [42]. Fifth, some hospitals may have adopted the robotic platform before entering the ACO model. If so, they may have experienced increased pressure to use it to maximize return on investment. While the financial incentives under the MSSP ACO model apply only to care provided to Medicare beneficiaries, we included all surgical cases in our analysis based on the prior evidence that provider behavior changes little in response to patient health insurance [58].

## Conclusion

In summary, our results indicate that hospital participation in later versions of the MSSP is associated with a moderate value improvement including more selective use of robotically assisted procedures and lower per patient spending. We also demonstrated that higher hospital volumes of robotic procedures correlated positively with higher hospital rankings. These findings suggest that hospitals that invest more in quality improvement and marketing are more likely to achieve Five-star CMS ranking and also more likely to invest in developing robotic capabilities. We also demonstrated that regardless of ACO status, the NYS hospitals experienced significant changes during the last decade, with increases in inpatient revenue and MSPB, and decreases in discharges. Given significant shortages of healthcare providers and the high prevalence of burn-out among surgeons and other hospital providers, quality improvement efforts should consider provider-level factors such as ergonomics, workload management, and access to adequate training and resources. These factors are crucial in reducing medical errors, improving efficiency, and ultimately enhancing both provider well-being and patient outcomes. As the US population shifts in distribution towards older ages, improving the value of surgical services is an important target for financial sustainability of the health care system and publicly funded programs.

## Supplementary Information

Below is the link to the electronic supplementary material.

Supplementary file1 (DOCX 28 KB)

## References

[1] Blumenthal D, Abrams M (2020) The affordable care act at 10 years—payment and delivery system reforms. N Engl J Med 382:1057–1063 32101657 10.1056/NEJMhpr1916092

[2] Berwick DM (2011) Making good on ACOs’ promise–the final rule for the Medicare shared savings program. N Engl J Med 365:1753–1756 22013899 10.1056/NEJMp1111671

[3] McWilliams JM, Landon BE, Chernew ME, Zaslavsky AM (2014) Changes in patients’ experiences in medicare accountable care organizations. N Engl J Med 371:1715–1724 25354105 10.1056/NEJMsa1406552 PMC4239654

[4] Nyweide DJ, Lee W, Colla CH (2020) Accountable care organizations’ increase in nonphysician practitioners may signal shift for health care workforce. Health Aff (Millwood) 39:1080–1086 32479221 10.1377/hlthaff.2019.01144

[5] Noble DJ, Casalino LP (2013) Can accountable care organizations improve population health?: should they try? JAMA 309:1119–1120 23512057 10.1001/jama.2013.592

[6] New York State Department of Health (2022) Data governance policy and procedure manual for data release: statewide planning and research cooperative system

[7] Dupree JM, Patel K, Singer SJ, West M, Wang R, Zinner MJ, Weissman JS (2014) Attention to surgeons and surgical care is largely missing from early medicare accountable care organizations. Health Aff (Millwood) 33:972–979 24889946 10.1377/hlthaff.2013.1300

[8] Modi PK, Kaufman SR, Borza T, Oliphant BW, Ryan AM, Miller DC, Shahinian VB, Ellimoottil C, Hollenbeck BK (2019) Medicare accountable care organizations and use of potentially low-value procedures. Surg Innov 26:227–233 30497340 10.1177/1553350618816594 PMC6503656

[9] Resnick MJ, Graves AJ, Buntin MB, Richards MR, Penson DF (2018) Surgeon participation in early accountable care organizations. Ann Surg 267:401–407 28338515 10.1097/SLA.0000000000002233

[10] Chukmaitov A, Harless DW, Bazzoli GJ, Muhlestein DB (2019) Preventable hospital admissions and 30-day all-cause readmissions: does hospital participation in accountable care organizations improve quality of care? Am J Med Qual 34:14–22 29848000 10.1177/1062860618778786

[11] Ryan AM, Krinsky S, Adler-Milstein J, Damberg CL, Maurer KA, Hollingsworth JM (2017) Association between hospitals’ engagement in value-based reforms and readmission reduction in the hospital readmission reduction program. JAMA Intern Med 177:862–868 28395006 10.1001/jamainternmed.2017.0518 PMC5800776

[12] Winblad U, Mor V, McHugh JP, Rahman M (2017) ACO-affiliated hospitals reduced rehospitalizations from skilled nursing facilities faster than other hospitals. Health Aff (Millwood) 36:67–73 28069848 10.1377/hlthaff.2016.0759 PMC5553196

[13] Navathe AS, Liao JM, Wang E, Isidro U, Zhu J, Cousins DS, Werner RM (2021) Association of patient outcomes with bundled payments among hospitalized patients attributed to accountable care organizations. JAMA Health Forum 2:e212131–e212131 35977188 10.1001/jamahealthforum.2021.2131 PMC8796940

[14] Hollingsworth JM, Nallamothu BK, Yan P, Ward S, Lin S, Colla CH, Lewis VA, Ayanian JZ, Hollenbeck BK, Ryan AM (2018) Medicare accountable care organizations are not associated with reductions in the use of low-value coronary revascularization. Circ Cardiovasc Qual Outcomes 11:e004492 29903936 10.1161/CIRCOUTCOMES.117.004492 PMC6005663

[15] Nathan H, Thumma JR, Ryan AM, Dimick JB (2019) Early impact of medicare accountable care organizations on inpatient surgical spending. Ann Surg 269:191–196 29771724 10.1097/SLA.0000000000002819 PMC7058185

[16] Schiavone MB, Kuo EC, Naumann RW, Burke WM, Lewin SN, Neugut AI, Hershman DL, Herzog TJ, Wright JD (2012) The commercialization of robotic surgery: unsubstantiated marketing of gynecologic surgery by hospitals. Am J Obstet Gynecol 207(174):e171-177 10.1016/j.ajog.2012.06.050 22835493

[17] Shen C, Gu D, Klein R, Zhou S, Shih YT, Tracy T, Soybel D, Dillon P (2020) Factors associated with hospital decisions to purchase robotic surgical systems. MDM Policy Pract 5:2381468320904364 32072012 10.1177/2381468320904364 PMC6997967

[18] Wright JD, Tergas AI, Hou JY, Burke WM, Chen L, Hu JC, Neugut AI, Ananth CV, Hershman DL (2016) Effect of regional hospital competition and hospital financial status on the use of robotic-assisted surgery. JAMA Surg 151:612–620 26886156 10.1001/jamasurg.2015.5508

[19] Carbonara U, Srinath M, Crocerossa F, Ferro M, Cantiello F, Lucarelli G, Porpiglia F, Battaglia M, Ditonno P, Autorino R (2021) Robot-assisted radical prostatectomy versus standard laparoscopic radical prostatectomy: an evidence-based analysis of comparative outcomes. World J Urol 39:3721–3732 33843016 10.1007/s00345-021-03687-5

[20] Huang Y, Chua TC, Maddern GJ, Samra JS (2017) Robotic cholecystectomy versus conventional laparoscopic cholecystectomy: a meta-analysis. Surgery 161:628–636 28011011 10.1016/j.surg.2016.08.061

[21] Marchand G, Taher Masoud A, Ware K, Govindan M, King A, Ruther S, Brazil G, Calteux N, Coriell C, Ulibarri H, Parise J, Arroyo A, Filippelli C, Loli H, Sainz K (2021) Systematic review and meta-analysis of all randomized controlled trials comparing gynecologic laparoscopic procedures with and without robotic assistance. Eur J Obstet Gynecol Reprod Biol 265:30–38 34418694 10.1016/j.ejogrb.2021.07.038

[22] Muaddi H, Hafid ME, Choi WJ, Lillie E, de Mestral C, Nathens A, Stukel TA, Karanicolas PJ (2021) Clinical outcomes of robotic surgery compared to conventional surgical approaches (laparoscopic or open): a systematic overview of reviews. Ann Surg 273:467–473 32398482 10.1097/SLA.0000000000003915

[23] Varghese A, Doglioli M, Fader AN (2019) Updates and controversies of robotic-assisted surgery in gynecologic surgery. Clin Obstet Gynecol 62:733–748 31524659 10.1097/GRF.0000000000000489 PMC6944326

[24] Sheetz KH, Nathan H, Dimick JB (2019) Patients’ perceptions of hospitals affiliated with America’s highest-rated medical centers. J Gen Intern Med 34:787–788 30632101 10.1007/s11606-018-4822-y PMC6544712

[25] Herrel LA, Yan P, Modi P, Adler-Milstein J, Ryan AM, Hollingsworth JM (2022) Association of medicare beneficiary and hospital accountable care organization alignment with surgical cost savings. JAMA Health Forum 3:e224817 36547947 10.1001/jamahealthforum.2022.4817 PMC9857079

[26] Childers CP, Maggard-Gibbons M (2018) Estimation of the acquisition and operating costs for robotic surgery. JAMA 320:835–836 30167686 10.1001/jama.2018.9219 PMC6142989

[27] Munoz E, Munoz W 3rd, Wise L (2010) National and surgical health care expenditures, 2005–2025. Ann Surg 251:195–200 20054269 10.1097/SLA.0b013e3181cbcc9a

[28] Lopez VH, Vatcheva K, Betancourt-Garcia MM, Dono A, Martinez RD, Forse RA (2022) Impact of accountable care organizations on acute cholecystitis outcomes in the Rio Grande Valley. Surgery 171:785–792 35034795 10.1016/j.surg.2021.08.069

[29] Modi PK, Kaufman SR, Caram ME, Ryan AM, Shahinian VB, Hollenbeck BK (2021) Medicare accountable care organizations and the adoption of new surgical technology. J Am Coll Surg 232(138–145):e132 10.1016/j.jamcollsurg.2020.10.016 PMC8030123 33122038

[30] Burstein MD, Myneni AA, Towle-Miller LM, Simmonds I, Gray J, Schwaitzberg SD, Noyes K, Hoffman AB (2022) Outcomes following robot-assisted versus laparoscopic sleeve gastrectomy: the New York State experience. Surg Endosc 36:6878–6885 35157123 10.1007/s00464-022-09026-y

[31] Hoffman AB, Myneni AA, Towle-Miller LM, Karim SA, Train AT, Burstein M, Schwaitzberg SD, Noyes K (2021) The early (2009–2017) experience with robot-assisted cholecystectomy in New York State. Ann Surg 274:e245–e252 34397456 10.1097/SLA.0000000000004932

[32] Centers for Medicare and Medicaid Services, Medicare Shared Savings Program (2022) Accountable care organization participants data dictionary

[33] Centers for Medicare and Medicaid Services (2022) Hospitals: about the data

[34] Centers for Medicare and Medicaid Services, Medicare Shared Savings Program (2022) Hospital provider cost report data dictionary

[35] Centers for Medicare and Medicaid Services (2022) Hospitals: Overall hospital quality star rating

[36] U.S. Department of Agriculture, Economic Research Service (2024) Rural-urban continuum codes

[37] Centers for Medicare and Medicaid Services, Open Payments (2023) Open payments: resources for reporting entities, Lists of teaching hospitals

[38] New York State Department of Health (2023) Data collection and usage: facility data. NYS Health Profiles

[39] Department of Health and Human Services, Centers for Medicare and Medicaid Services (2022) NPI: What you need to know

[40] SAS Institute Inc (2023) SAS/STAT 153 user’s guide. SAS Institute Inc, Cary

[41] Herrel LA, Norton EC, Hawken SR, Ye Z, Hollenbeck BK, Miller DC (2016) Early impact of medicare accountable care organizations on cancer surgery outcomes. Cancer 122:2739–2746 27218198 10.1002/cncr.30111 PMC4992435

[42] Kaye DR, Luckenbaugh AN, Oerline M, Hollenbeck BK, Herrel LA, Dimick JB, Hollingsworth JM (2020) Understanding the costs associated with surgical care delivery in the medicare population. Ann Surg 271:23–28 30601252 10.1097/SLA.0000000000003165 PMC6586534

[43] Intuitive Surgical, Inc. (2021) Annual report

[44] Sheetz KH, Claflin J, Dimick JB (2020) Trends in the adoption of robotic surgery for common surgical procedures. JAMA Netw Open 3:e1918911 31922557 10.1001/jamanetworkopen.2019.18911 PMC6991252

[45] Kite-Powell J (2021) Surgical robotics are here to stay. Forbes Forbes Media, Jersey city

[46] McWilliams JM, Hatfield LA, Chernew ME, Landon BE, Schwartz AL (2016) Early performance of accountable care organizations in medicare. N Engl J Med 374:2357–2366 27075832 10.1056/NEJMsa1600142 PMC4963149

[47] Mukherjee P, Khadra M, Merrett N, Rawstron E, Richardson A, Sutherland K, Levesque JF (2022) Value-based care in surgery: implications in crisis and beyond. ANZ J Surg 92:646–648 35434954 10.1111/ans.17501 PMC9324063

[48] Pham H, Ginsburg PB (2018) Payment and delivery-system reform—the next phase. N Engl J Med 379:1594–1596 30230961 10.1056/NEJMp1805593

[49] Mederos MA, Jacob RL, Ward R, Shenoy R, Gibbons MM, Girgis MD, Kansagara D, Hynes D, Shekelle PG, Kondo K (2022) Trends in robot-assisted procedures for general surgery in the veterans health administration. J Surg Res 279:788–795 35970011 10.1016/j.jss.2022.06.055

[50] McWilliams JMC, Alice JC (2020) Understanding the latest ACO “savings”: curb your enthusiasm and sharpen your pencils—Part 1. Health Aff. 10.1377/forefront.20201106.71955

[51] Colla CH, Lewis VA, Tierney E, Muhlestein DB (2016) Hospitals participating In ACOs tend to be large and urban, allowing access to capital and data. Health Aff (Millwood) 35:431–439 26953297 10.1377/hlthaff.2015.0919 PMC4838188

[52] Burke GC (2019) New York’s Medicare accountable care organizations improve performance in year 5 of the Medicare Shared Savings Program.

[53] Joynt K, Nguyen N, Samson LW, Lechner A, Ogunwumiju O (2016) ASPE Issue brief: Rural hospital participation and performance in value-based purchasing and other delivery system reform initiatives. Department of Heath and Human Services, Office of the Assistant Secretary for Planning and Evaluation

[54] (2020) Healthcare: The state of horizontal consolidation. The hospital association of New York State intelligence report

[55] Schoenfeld AJ, Sturgeon DJ, Blucher JA, Haider AH, Kang JD (2019) Alterations in 90 day morbidity, mortality, and readmission rates following spine surgery in medicare accountable care organizations (2009–2014). Spine J 19:8–14 30010045 10.1016/j.spinee.2018.06.367

[56] United States Congressional Budget Office (2022) The long-term budget outlook Congressional Budget Office, Washington

[57] Wu FM, Shortell SM, Lewis VA, Colla CH, Fisher ES (2016) Assessing differences between early and later adopters of accountable care organizations using taxonomic analysis. Health Serv Res 51:2318–2329 26927979 10.1111/1475-6773.12473 PMC5134136

[58] Wilensky G (2016) Changing physician behavior is harder than we thought. JAMA 316:21–22 27380330 10.1001/jama.2016.8019

