# Supplementary material for: Association between hospital participation in Medicare Shared Savings Program and hospital use of robotic surgical approach
Source: Surg Endosc. 2025 Mar 21;39(5):2982–93. doi: 10.1007/s00464-025-11656-x (PMC12041123; doi:10.1007/s00464-025-11656-x)
Supplement: Supplementary file 1 — Supplementary file1 (DOCX 28 KB) [file 464_2025_11656_MOESM1_ESM.docx]

| **Supplemental Table 1.** ICD-9, ICD-10, and CPT Procedure Codes | | | |
| --- | --- | --- | --- |
| **Procedure Type** | **ICD-9 codes** | **ICD-10 codes** | **CPT codes** |
| Robotic | 1741, 1742, 1743, 1744, 1745, 1749 | 8E0W0CZ, 8E0W3CZ, 8E0W4CZ, 8E0W7CZ, 8E0W8CZ, 8E0WXCZ, 8E090CZ, 8E093CZ, 8E094CZ, 8E097CZ, 8E098CZ, 8E09XCZ | S2900 |
| Cholecystectomy | 5121, 5122, 5124 | 0FB44ZX, 0FB44ZZ, 0FB48ZX, 0FB48ZZ, 0FT44ZZ, 0FB40ZZ, 0FB43ZZ, 0FT40ZZ | 47562, 47563, 47600, 47601, 47602, 47603, 47604, 47605, 47606, 47607, 47608, 47609, 47610, 47611, 47612, 47613, 47614, 47615, 47616, 47617, 47618, 47619, 47620 |
| Bariatric | 436, 437, 445, 4382, 4389, 4431, 4438, 4439, 4468, 4469, 4495, 4496, 4497, 4498, 4499, 4551, 4591, | 0DB64Z3, 0DB60Z3, 0DB60ZZ, 0DB67Z3, 0D16079, 0D1607A, 0D160J9, 0D160JA, 0D160K9, 0D160KA, 0D160Z9, 0D160ZA, 0D160ZB, 0D16479, 0D1647A,  0D164J9, 0D164JA, 0D164K9, 0D164KA, 0D164Z9, 0D164ZA, 0D190Z9,  0D190ZA,  0D190ZB, 0D1A0ZA,  0D1A0ZB, 0D1BOZB, 0DB60Z3,  0DB60ZX, 0DB60ZZ, 0DB63Z3, 0DB63ZX, 0DB63ZZ, 0DB64Z3, 0DB64ZX, 0DB64ZZ, 0DB67Z3, 0DB67ZX, 0DB80ZZ, 0DB90ZZ, 0DBB0ZZ, 0DP643Z, 0DP64CZ, 0F190Z3, 3E0G4GC, 0DV67ZZ, 0DV68ZZ, 0DV64ZZ, 0DV64DZ, 0DV64CZ, 0DV63ZZ, 0DV63DZ, 0DV63CZ, 0DV60ZZ, 0DV60DZ, 0DV60CZ, 0DQ68ZZ, 0DQ67ZZ, 0DQ64ZZ, 0DQ63ZZ, 0DQ60ZZ, 0D168ZA, 0D168Z9, 0D168KA, 0D168K9, 0D168JA, 0D168J9, 0D1687A, 0D16879 | 43633, 43644, 43645, 43659, 43770, 43772, 43773, 43774, 43775, 43842, 43843, 43845, 43846, 43847, S2082, S2085 |
| Gynecologic | 65, 66, 67, 68, 69, 70, 71 | 0U | 56, 57, 58 |
| Genitourinary | 55, 56, 57, 58, 59, 60, 61, 62, 63, 64 | 0T, 0V | 50, 51, 52, 53, 54, 55 |
